# Partizipation und Ko-Kreation in der Implementierungsforschung

**DOI:** 10.1007/s00103-025-04085-7

**Published:** 2025-06-19

**Authors:** Anja Zscheppang, Christiane Falge, Silke Betscher, Anna Köster-Eiserfunke, Jonas Fiedler, Claudia Czernik, Claudia Hoevener, Anna Kuehne

**Affiliations:** 1 Professur Öffentliche Gesundheit, Zentrum für Evidenzbasierte Gesundheitsversorgung (ZEGV), Universitätsklinikum und Medizinische Fakultät Carl Gustav Carus, TU Dresden, Fetscherstr. 74, 01307 Dresden, Deutschland; 2 Professur für Gesundheit und Diversity, Stadtteillabor Bochum, Hochschule Bochum, Bochum, Deutschland; 3 Professur für Gemeinwesenarbeit, Community Development und Macro Social Work Department Soziale Arbeit, HAW, Hamburg, Deutschland; 4 Poliklinik Veddel, Hamburg, Deutschland; 5 Alice Salomon Hochschule, University of Applied Sciences, Berlin, Deutschland

**Keywords:** Implementierungswissenschaft, Partizipative Gesundheitsforschung, Forschungsmethoden, Public Health, Evidenzbasierte Praxis, Implementation science, Community-based participatory research, Evidence-based practice, Capacity building, Public health, Germany

## Abstract

Ziel der Implementierungsforschung ist es evidenzbasierte Interventionen aus der Wissenschaft in die Praxis zu transferieren. Für einen erfolgreichen Transfer braucht es die Akzeptanz der Interventionen durch die Nutzenden und Umsetzenden. In Forschungsprozessen finden vermehrt partizipative Ansätze Berücksichtigung, in welchen verschiedene Akteur:innen an der Forschung und Implementierung beteiligt werden und damit die Akzeptanz von Maßnahmen erhöht wird. Partizipierende kommen je nach Intervention aus den Bereichen der Nutzenden, Wissenschaft, Versorgung, Politik oder Finanzierung. Eine besondere Rolle nehmen in partizipativen Prozessen die Bürger:innen und Patient:innen ein. Die partizipative Implementierungsforschung ist ein kollaborativer Ansatz, der die Implementierungsforschung mit der Ko-Kreation von Wissen verbindet, indem die Beteiligten systematisch in den Forschungsprozess und darüber hinaus eingebunden werden. Somit lassen sich nutzer:innenorientierte, passgenaue, lebensweltorientierte Interventionen der Gesundheitsförderung und der Gesundheitsversorgung wissenschaftlich unter Realbedingungen entwickeln und dauerhaft in die Praxis überführen. Dieser Übersichtsartikel beleuchtet den Stand der partizipativen Implementierungsforschung in Deutschland und geht auf Konzepte und Rahmenbedingungen der partizipativen Implementierungsforschung ein. Dafür werden Projekte aus den Bereichen Patientenversorgung, Gesundheitsförderung und dem Setting Kommune beschrieben. Zwei Beispiele der langfristigen Beteiligung von Bürger:innen eines Stadtteils an Forschung in den Stadtteillaboren Bochum und Hamburg zeigen, wie eine kontinuierliche Partizipation und Ko-Kreation in der Implementierungsforschung im Bereich der Prävention, Gesundheitsförderung und Gesundheitsversorgunggelingen kann.

Eine englische Übersetzung des Artikels steht im Online-Material zur Verfügung.

**Zusatzmaterial online:**

Zusätzliche Informationen sind in der Online-Version dieses Artikels (10.1007/s00103-025-04085-7) enthalten.

## Abstract (en)

The aim of implementation research is to transfer evidence-based interventions from research into practice. Successful transfer requires acceptance of the interventions by users and implementers. Research processes are increasingly focussing on participatory approaches in which various stakeholders are involved in research and implementation, thereby increasing the acceptability of interventions. Depending on the intervention, participation may draw from users, research, care providers or policy and funding bodies. Citizens and patients play a crucial role in participatory processes. Participatory implementation research is a collaborative approach that combines implementation research with the co-creation of knowledge by systematically involving participants in the research process and beyond. In this way, user-centred, tailor-made, lifeworld-oriented interventions in health promotion and healthcare can be scientifically developed under real-life conditions and permanently transferred into real-world practice. This review article examines the status of participatory implementation research in Germany and outlines the concepts and framework of participatory implementation research. To this end, projects from the fields of patient care, health promotion and the community setting are described. Two examples of the long-term participation of citizens of a neighbourhood in research conducted in the urban district laboratories in Bochum and Hamburg show how continuous participation and co-creation in implementation research for prevention, health promotion and health care can succeed.

An English full-text version of this article is available at SpingerLink as Supplementary Information.

## Einleitung

Partizipative Ansätze in der Implementierungsforschung ermöglichen die gemeinsame Entwicklung relevanter Forschungsziele und Indikatoren, erhöhen die Praxisnähe der Methodik und fördern Netzwerkbildung, Empowerment sowie Kapazitätenaufbau. Durch die Integration von partizipativen Ansätzen kann der Implementierungsprozess gestärkt und ein Beitrag zur Nachhaltigkeit der Umsetzung geleistet werden. In der folgenden narrativen Übersicht geben wir einen Überblick über die Ansätze der Implementierungsforschung, der partizipativen Forschung, die Möglichkeiten der Verbindung beider Forschungsansätze sowie Beispiele partizipativer Implementierungsforschung in Deutschland.

## Was ist Implementierungsforschung?

Während für zahlreiche Public-Health-Maßnahmen die Wirksamkeit wissenschaftlich erwiesen ist, dauert der Wissenschafts-Praxis-Transfer dieser Maßnahmen häufig viele Jahre oder scheitert unter realen Bedingungen [1]. Diese fehlende Integration von evidenzbasierten Anwendungen in die Praxis führt zu Unter‑, Über- und Fehlversorgung [2]. Die Übersetzung von wissenschaftlichen Erkenntnissen in die praktische Umsetzung ist das Forschungsfeld der Implementierungswissenschaft [3, 4].

Implementierungsforschung untersucht, wie Interventionen, deren grundsätzliche Wirksamkeit bereits erwiesen ist, in angemessener Weise in der Praxis implementiert werden können [3, 5, 6]. Im Fokus steht die Identifizierung von fördernden und hemmenden Faktoren der Implementierung. Ein Hauptaugenmerk liegt zudem auf der Nachhaltigkeit, also einer langfristigen Durchführung von Interventionen über die Implementierungsphase hinaus [3, 5, 6]. Zu den umsetzungsbezogenen Faktoren, die in der Implementierungsforschung untersucht werden, gehören [7]:

- Akzeptanz und Angemessenheit der Intervention,
- Machbarkeit, Übernahme und Ausmaß der Nutzung in der Praxis,
- Wiedergabetreue der Originalintervention,
- Nachhaltigkeit und
- Kosten der Intervention.

Um Implementierungsprozesse, Rahmenbedingungen und Outcomes systematisch zu erfassen, werden in der Implementierungsforschung verschiedene Theorien, Modelle und Frameworks (TMFs) angewendet [8]. Das „Consolidated Framework for Implementation Research“ (CFIR) stellt einen Ansatz dar, um kontextbezogene Faktoren systematisch zu erfassen [9]. Das Rahmenmodell des CFIR fasst in 5 Dimensionen potenzielle Faktoren für den Transfer zusammen [9]: Merkmale der Intervention, inneres Setting (innerhalb der Organisation), äußeres Setting (außerhalb der Organisation, z. B. gesetzliche Rahmenbedingungen und Marktsituation), Merkmale beteiligter Personen und Prozess bei der Implementierung.

Der Artikel von Weishaar et al. in diesem Themenheft bietet einen umfassenden Überblick über die Implementierungswissenschaft im Public-Health-Bereich in Deutschland sowie einen Einblick in weitere TMFs und Anwendungsbeispiele.

Weitere Eigenschaften der Implementierungsforschung sind die interdisziplinäre Kollaboration, die Identifizierung von kontextspezifischen Erfolgsfaktoren, die Erforschung von Möglichkeiten des Aufbaus von Kapazitäten zur Verstetigung, die Entwicklung von Netzwerken, die iterative Verbesserung und das kontinuierliche Lernen sowie die Erprobung von Möglichkeiten der Skalierung [5]. Die Einbindung von Wissenschaftler:innen, Versorgenden, Nutzenden sowie Akteur:innen aus Politik und Finanzierung in die Implementierungsforschung kann dabei von entscheidender Bedeutung sein, um die Relevanz und Akzeptanz der Intervention zu erhöhen [5, 7].

## Was sind partizipative Forschung und Ko-Kreation?

Stummer (2023) beschreibt, wie die Implementierungsforschung von partizipativen Ansätzen profitieren kann: „Durch die Förderung von Partnerschaften zwischen Forschern, Praktikern, politischen Entscheidungsträgern und Gemeinden kann die Implementierungsforschung den Austausch von Wissen, Erfahrungen und Ressourcen erleichtern, was letztlich zu wirksameren Maßnahmen und Verbesserungen der öffentlichen Gesundheit führt“ [5].

Die Integration von Ansätzen partizipativer Gesundheitsforschung (PGF) kann die Implementierungswissenschaft in den Bereichen Gesundheitsförderung und Gesundheitsversorgung ergänzen und einen Rahmen für die strukturierte Beteiligung unterschiedlicher Akteur:innen in den Forschungsprozess bieten. Partizipative Gesundheitsforschung unterstützt dabei, die Lücke zwischen Forschungsergebnissen und praktischer Anwendung zu überbrücken, und kann so dazu beitragen, dass die Maßnahmen nicht nur evidenzbasiert, sondern auch kontextbezogen und nachhaltig sind [10]. Die Einbindung der Nutzenden erleichtert es, die Gesundheit von Gruppen, die von Gesundheitsförderung, Gesundheitsversorgung und Forschung häufig besonders schlecht erreicht werden, zu verbessern [5, 11, 12].

Partizipative Gesundheitsforschung (PGF) ist gekennzeichnet durch die direkte Beteiligung von Personen am Forschungsprozess, deren Arbeits- oder Lebensverhältnisse, Gesundheitszustand oder Bedarfe Gegenstand der Forschung sind [13, 14]. Die Beteiligung kann hierbei alle Menschen einbeziehen, die durch die Forschung oder den Forschungsgegenstand betroffen sind. Partizipation schließt Finanzierende, politische Entscheidungstragende, Praktiker:innen, z. B. Pflegende, Ärzt:innen und andere Heilberufler:innen, Vertretende von Patient:innen oder Bürger:innen ein [15, 16]. Für die Implementierungswissenschaft sind dabei sowohl die Zielgruppe der Intervention als auch die Umsetzenden von Relevanz.

In der PGF wird die Beteiligung als eine Forschungskollaboration verstanden, die sich im Idealfall über den gesamten Forschungsprozess erstreckt. Sie beginnt mit der gemeinsamen Festlegung der Forschungsfrage, gefolgt von der gemeinsamen Methodenfestlegung, Datenerhebung und Datenauswertung. PGF beinhaltet auch die gemeinsame Interpretation und Entwicklung von Empfehlungen [14]. Partizipative Forschung ist damit keine Festlegung auf eine bestimmte Form der Datenerhebung – also keine Festlegung auf qualitative oder quantitative Methodik oder bestimmte TMFs –, sondern entspricht eher einer Herangehensweise an den Forschungsprozess [14, 17, 18]. Diese Herangehensweise ist charakterisiert durch Forschung, die eingebettet ist in den lokalen Kontext mit partizipativen Entscheidungen und einem Fokus auf den kollektiven Forschungsprozess, in dem zumindest ein Teil der Entscheidungsmacht von den Wissenschaftler:innen an die übrigen beteiligten Akteur:innen übertragen wird [14, 19].

Es gibt verschiedene Schulen und Ausrichtungen der PGF, die unterschiedlich bezeichnet werden (z. B. Action Research, Community-based Participatory Research), die sich alle in der Regel durch eine Kooperation von Akteur:innen, die in dem beforschten Bereich aktiv sind oder betroffen sind (Nutzer:innen, Bürger:innen, Personal, Praktiker:innen etc.) und Wissenschaftler:innen auszeichnen sowie durch eine Verknüpfung von Wissensproduktion mit der Entwicklung neuer Handlungsoptionen oder Interventionen, die Arbeits- oder Lebensverhältnisse der Beteiligten verbessern [14]. Im Rahmen der PGF kommt es zu einer Ko-Kreation, also der gemeinsamen Erschaffung von Wissen im Rahmen eines gemeinsamen dialogischen Forschungsprozesses. Häufig wird dieser durch ein Forschungsteam, bestehend aus Vertreter:innen aller beteiligten Gruppen, gesteuert [20–22]. Ko-Kreation dient der kollaborativen und gleichberechtigten Wissensgenerierung. Die Beteiligten sind bereit, ihr Wissen, ihre Fähigkeiten und Ressourcen zu teilen. Es finden eine gemeinsame Planung, Gestaltung, Erprobung und Umsetzung der Intervention statt [23]. Ko-Kreation zielt auf die Verringerung von Ungleichheiten ab und kann nachhaltige Veränderungen ermöglichen [19].

Die direkte Beteiligung am Forschungsprozess ermöglicht die Entwicklung einer angemessenen, kontextorientierten und lokal akzeptierten Forschung und Intervention. Zudem ist die PGF eine gemeinschaftliche Anstrengung, die darauf abzielt, Netzwerke aufzubauen, das gegenseitige Lernen zu ermöglichen, relevante Empfehlungen zu entwickeln und so zu Nachhaltigkeit beizutragen [14, 22].

Partizipative Forschungsprozesse können sich in der Qualität der Partizipation, der Dauer der Partizipation und den Stufen der Partizipation unterscheiden. Hinsichtlich der Qualität der Partizipation wurden verschiedene Gütekriterien aufgestellt, sie umfassen unter anderem die Einbeziehung des Systems, in dem die Forschung stattfindet, sowie die Wertschätzung des Prozesses als einen kollektiven Lernprozess [19]. Die Beteiligung der Akteur:innen kann über den Forschungsprozess und für verschiedene Gruppen variieren. Im Stufenmodell der Partizipation nach Wright werden Vorstufen der Partizipation, wie Information, Anhörung oder Einbeziehung der Akteur:innen, die in dem beforschten Bereich aktiv sind oder betroffen sind, von tatsächlicher Partizipation in Form von Mitbestimmung, teilweiser Entscheidungskompetenz, Entscheidungsmacht oder gar Selbstorganisation unterschieden [21]. Die International Collaboration for Participatory Health (ICPHR) sowie das Netzwerk für partizipative Gesundheitsforschung (PartNet) bieten zu Qualitätsmerkmalen, Prozessgestaltung und Vernetzung vielfältige Materialien.^1^,^2^

Partizipative Forschung verbessert sowohl den Aufbau von Kapazitäten, d. h. Strukturen und Kompetenzen, für die Durchführung als auch die Nachhaltigkeit und Qualität von Interventionen [24, 25].

## Wie kann partizipative Forschung die Implementierungsforschung ergänzen?

„If we want more evidence-based practice, we need more practice-based evidence“ [26].

Das Ziel, wissenschaftliche Erkenntnisse unter Realbedingungen zu produzieren, eint die PGF und die Implementierungsforschung [27]: Partizipative Gesundheitsforschung legt den Schwerpunkt auf die Beteiligung der Betroffenen und Umsetzenden mit dem Ziel, verschiedene Blickwinkel in der Forschung zu berücksichtigen und so die Passung für den Kontext, Akzeptanz und Nachhaltigkeit zu erhöhen. Dasselbe Ziel verfolgt die Implementierungswissenschaft. Sie zielt ebenfalls darauf ab, Erkenntnisse kontextangepasst und langfristig in die Praxis umzusetzen [23, 27, 28].

Einige Autor:innen beschreiben partizipative Forschungsansätze als zentrale methodische Basis für Implementierungsforschung, die dazu beiträgt, die lokale Wirksamkeit zu erhöhen und bessere verallgemeinerbare Evidenz zu erzeugen [29–31]. Auch in Deutschland gibt es im Rahmen des Deutschen Netzwerks für Versorgungsforschung (DNVF) eine Arbeitsgruppe zu partizipativer Versorgungsforschung, in der auch partizipative Methoden der Implementierungswissenschaft beleuchtet werden.^3^

### Praktische Umsetzung der partizipativen Gesundheitsforschung in der Implementierungswissenschaft

Partizipative Implementierungswissenschaft wird international als Ansatz in der Implementierungswissenschaft beschrieben. Sie ist ein kollaborativer Forschungsansatz, der Wissenschaftler:innen, Betroffene und Umsetzende einbezieht, um evidenzbasierte Interventionen kontextadaptiert in die Praxis zu integrieren. Der partizipative Ansatz zielt darauf ab, die Gesundheit der Individuen in ihren Lebenswelten zu verbessern und die gesundheitliche Ungleichheit zu verringern [11, 32]. Im Gegensatz zu traditionellen Top-down-Modellen betonen die partizipativen Ansätze in der Implementierungsforschung die Koproduktion von Wissen durch einen relationalen und nichtlinearen Prozess. Die PGF kann die Passfähigkeit von Implementierungsstrategien verbessern, indem sie unmittelbares Feedback und iterative Verbesserungen ermöglicht, die für eine erfolgreiche Umsetzung in verschiedenen Settings entscheidend sind [11]. Partizipative Ansätze können während des gesamten Forschungsprozesses der Implementierungsforschung angewandt werden [11]. Die partizipative Implementierungswissenschaft berücksichtigt die Gestaltung von Netzwerken und die Entwicklung von Informationsformaten, die auf die Beteiligten zugeschnitten sind [11]. Durch die Einbeziehung von Methoden der PGF kann die Implementierungswissenschaft die Zeitspanne zwischen wissenschaftlicher Bestätigung der Wirksamkeit von Interventionen und deren Umsetzung in die Praxis verkürzen [28].

### Bandbreite und Variationen der partizipativen Gesundheitsforschung in der Implementierungsforschung

Tatsächlich beschreiben zahlreiche Autor:innen partizipative Ansätze in der Implementierungsforschung [10, 23, 28, 32–34]. Diese Ansätze unterscheiden sich hinsichtlich a) der Art und Anzahl der Akteur:innen, die eingebunden werden, b) hinsichtlich der Tiefe der Mitbestimmung (also der Stufe der Partizipation und der damit verbundenen Machtübergabe) sowie c) in ihrer Kontinuität und Dauer der Einbindung [23, 27, 29, 35, 36].

Die Einbindung der Beforschten erfolgt häufig projektspezifisch und ist zum Teil auf Einzelaspekte des Forschungsprozesses begrenzt. Partizipative Forschung in der Implementierungswissenschaft kann sowohl in der Forschungsplanung – etwa bei der Festlegung des (gesundheitlichen) Outcomes – als auch bei der Festlegung des Forschungsdesigns, der Interventionsgestaltung und der Dateninterpretation zum Einsatz kommen. Sie ist in allen Phasen des Forschungsprozesses oder auch darüber hinaus in Form langfristiger Forschungspartnerschaften möglich [10, 11, 23, 27, 37]. Einige Forschungspartnerschaften sind themenspezifisch und fokussieren auf ein bestimmtes Forschungsanliegen, während langfristige themenoffene Partnerschaften zwischen Universitäten, Praktiker:innen und Bürger:innen die gemeinsame Prioritätensetzung sowie Entwicklung von Implementierungsstrategien erlauben [37]. Eine besonders umfangreiche und kontinuierliche Form der PGF stellt „Community-Engaged Dissemination and Implementation Research“ (CEDI) [31] dar, die sich durch langfristige Partnerschaften zwischen Forschenden und Communitys, ein kontinuierliches Engagement der Community sowie eine gemeinsame Entwicklung von Projekten auszeichnet [31, 36]. In den US-amerikanischen Clinical and Translational Science Institutes findet Community Engagement im Sinne einer langfristigen kontinuierlichen Partnerschaft auf Augenhöhe bereits statt und wird zunehmend für Implementationsforschung genutzt [34].

**Abb. 1:**
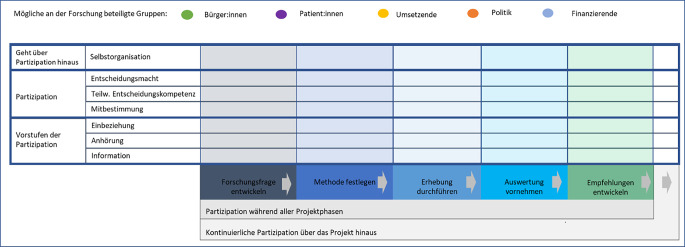
Visualisierung von Partizipationsmöglichkeiten in der partizipativen Gesundheitsforschung: Partizipation unterschiedlicher Akteur:innen, auf verschiedenen Partizipationsstufen, während des Forschungsprozesses (eigene Abbildung angelehnt an Krieger T, Nellessen-Martens, G (2023) [38] und Wright M (2021) [12]). Dabei sind links die Stufen der Partizipation nach Wright M (2021) dargestellt. In den farbigen Spalten sind die verschiedenen Phasen des Forschungsprozesses abgetragen. Die bunten Punkte verdeutlichen jeweils eine:n Akteur:in. Diese können mehrfach in die Tabelle eingetragen werden, um zu verdeutlichen, wer wann mit welcher Stufe der Partizipation in den Forschungsprozess eingebunden ist

Zusammenfassend kann die Anwendung von PGF in der Implementierungsforschung unterschiedliche Akteur:innen einbeziehen, in unterschiedlichen Phasen des Forschungsprozesses genutzt werden und verschiedene Stufen der Beteiligung beinhalten ([38]; Abb. 1).

## Beispiele partizipativer Ansätze in der Implementierungsforschung in Deutschland

In Deutschland existieren viele Projekte, die partizipative Ansätze in der Implementierungsforschung wählen. Nicht immer wird in den Projekten der partizipative Forschungsansatz bzw. Ko-Kreation und/oder die Zugehörigkeit zur Implementierungsforschung explizit benannt. Wir stellen im Folgenden ausgewählte Projekte vor, die:

- partizipative Forschungsmethoden im Sinne einer Ko-Kreation mit Abgabe von Entscheidungsmacht an Akteur:innen, die in dem beforschten Bereich aktiv sind oder betroffen sind, beinhalten und
- die sich mit einem Inhalt der Implementierungsforschung beschäftigten, wie der Erforschung der Integration einer Maßnahme, deren Wirksamkeit schon bewiesen ist, in einem bestimmten Kontext/für eine bestimmte Gruppe.

Im Folgenden wird exemplarisch auf einzelne partizipative Projekte aus dem Bereich der Implementierungswissenschaft eingegangen, um die Bandbreite der partizipativen Forschung in Deutschland zu veranschaulichen. Die Forschungsprojekte unterscheiden sich in der Qualität der Partizipation, ihrer Kontinuität und in der Intensität der Partizipation. Sie adressieren unterschiedliche Gruppen und verwenden unterschiedliche Forschungsmethoden. Allen Projekten ist gemein, dass sie die Übertragung einer Intervention mit nachgewiesenem Gesundheitsbenefit in die Praxis beforschen. In der Literatur haben wir kein Beispiel in Deutschland gefunden, in dem partizipative Forschungsmethoden angewandt wurden und auf strukturierte TMFs der Implementierungsforschung zurückgegriffen worden ist.

### Patient:innenbeteiligung in der Implementierungsforschung in der medizinischen Versorgung

Ansätze der partizipativen Forschung finden sich in verschiedenen Fachgebieten der medizinischen Versorgung. Hervorzuheben sind entsprechende Ansätze der deutschen Rheuma-Liga^4^, die dort systematisch gefördert werden und sich für Forschungsprojekte der Implementierungsforschung besonders eignen könnten, da die Lebenswelt der Patient:innen in Bezug auf ihre Versorgung erforscht wird und die Ergebnisse in der Praxis für die Patient:innen bedeutend sind.^4^ Zudem gibt es Ansätze der systematischen Einbindung von Patient:innen und Bürger:innen in den Netzwerken der allgemeinmedizinischen Praxen. Die Beteiligung findet in Patient:innenbeiräten oder in Bürgerforen statt und fokussiert die Studienkonzeption, den Transfer in die Praxis sowie die Dissemination der Ergebnisse [39].

### Partizipation in der Gesundheitsförderung am Beispiel Bewegungsförderung

*Capital4Health *– „Capabilites for active lifestyle: An interactive knowledge-to-action research network for health promotion“ – ist ein Forschungsverbund, welcher in Teilprojekten die Erforschung und Entwicklung von Handlungsmöglichkeiten für einen gesunden Lebensstil während der gesamten Lebensspanne fokussiert. Diese Teilprojekte verfolgen partizipative Ansätze, indem sie durchführende Akteur:innen sowie die angesprochene Bevölkerungsgruppen in den Forschungsprozess einbeziehen.^5^ Mittels „kooperativer Planung“ werden verschiedene Altersgruppen in unterschiedlichen Settings in die Erarbeitung von Maßnahmen bzw. konkreten Handlungsstrategien zur Bewegungsförderung in sogenannten Planungsgruppen aktiv eingebunden [40, 41].

Das Teilprojekt „Health.edu“ (2015–2018) zielte auf die nachhaltige Entwicklung sportbezogener Gesundheitskompetenz von Schüler:innen ab. In kooperativen Planungsgruppen an 4 Schulen im Setting Sportunterricht waren Sportlehrkräfte, Schulleitung, Wissenschaftler:innen und Schüler:innen an mehreren Treffen (über 12 bis 18 Monate) beteiligt, um ein Curriculum in den Sportunterricht zu implementieren. Parallel dazu fanden im Setting Sportlehrerbildung ebenfalls Treffen mit Dozierenden, Seminarlehrkräften, Koordinator:innen der Lehrerbildungsphasen, Wissenschaftler:innen sowie Studierenden statt. Die Ergebnisse zeigen, dass die Intervention im Sportunterricht zur Stärkung der Gesundheitskompetenz der Schüler:innen erfolgreich war und signifikant bessere Ergebnisse als in den Kontrollschulen erreicht wurden. Die Methode der „kooperativen Planung“ schätzten die Teilnehmenden als einen „lohnenden Prozess“ ein, der in einem wertschätzenden Setting stattfand. Durch die Beteiligung der Schüler:innen konnten deren Sichtweisen auf Sportunterricht und damit deren Expertise in der Maßnahmenplanung berücksichtigt werden. So wurde in dem Projekt der Sportunterricht als bewegungsfördernde Gesundheitsintervention im Sinne partizipativer Implementierungsforschung kontextbezogen angepasst und im Forschungsprozess zudem ein größeres Verständnis von Gesundheit und Bewegung erzielt [41]. Es wurden Aspekte der Akzeptanz, Machbarkeit und Übernahme im Rahmen des Projektes erforscht.

### Partizipation in der Kommune – kommunale Gesundheitsstrategien

Unter dem Dach des Forschungsverbundes PartKommPlus „Forschungsverbund für gesunde Kommunen“ fanden sich 7 partizipative Forschungsprojekte, die das Ziel hatten, eine gesunde Lebensweise durch kommunale oder betriebliche Angebote zu unterstützen.^6^ In einigen der geförderten Projekte wurden Gesundheits- und Public-Health-Interventionen, deren Wirksamkeit bereits erwiesen ist, mit Ansätzen der Implementierungsforschung verknüpft und so hemmende und fördernde Faktoren für die Implementierung identifiziert. Zudem wurden lebensweltlich orientierte nutzer:innenangepasste Interventionen entwickelt.

Das partizipative Forschungsprojekt GESUND! untersuchte als eines der PartKommPlus-Projekte die Gesundheitsförderung für und mit Menschen mit Lernschwierigkeiten [42]. Es bestand aus mehreren Teilprojekten, die jeweils Maßnahmen der Gesundheitsförderung auf die Zielgruppe der Menschen mit Lernschwierigkeiten angepasst haben. Es wurden dabei im Rahmen partizipativer Forschungsprojekte, die sowohl quantitative Erhebungen als auch qualitative Verfahren und Photo-Voice-Methodik verwendet haben, Interventionen aus den Bereichen gesunde Ernährung, Reduktion von Lärmbelastung, betriebliche Gesundheitsförderung in Werkstätten für Menschen mit Lernschwierigkeiten sowie Gesundheitsbildung in ihrer Implementierung beforscht und zum Teil langfristig etabliert [41].

Ein weiteres Projekt aus dem PartKommPlus-Verbund ist das Projekt KEG (Kommunale Entwicklung von Gesundheitsstrategien: Wissenschaft und Praxis im Dialog), ein partizipatives Projekt, das der Frage nachgeht, wie die Entwicklung und Implementierung von kommunalen Gesundheitsstrategien gelingen können [43]. Es kamen Umsetzende der Gesundheitsförderung und junge Bewohner:innen des Stadtteils zusammen und nutzten die Methode „Appreciative Inquiry“ (dt. „wertschätzende Befragung“), um in einem qualitativen Forschungsverfahren Interviews mit Akteur:innen sowie Bewohner:innen des Stadtteils zu führen. In dem Rahmen wurde durch die Wissenschafts-Praxis-Partnerschaft ein Framework entwickelt und evaluiert, um kommunale Gesundheitsstrategien lokal kontextangepasst zu entwickeln und zu implementieren [43].

### Kontinuierliche partizipative Forschung im Rahmen von Stadtteillaboren

**Abb. 2:**
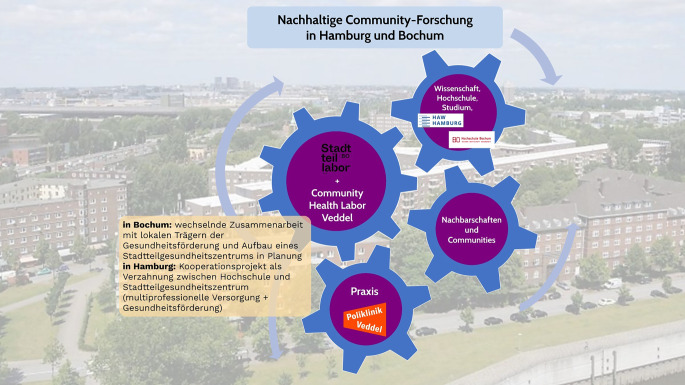
Struktur der Stadtteillabore Bochum und Community-Health-Labor (CHL) Veddel. Eigene Abbildung

Der methodisch-konzeptionelle Ansatz eines Stadtteillabors zeichnet sich durch eine enge Verbindung von Hochschule, Praxisträgern und Nachbarschaften aus. Abb. 2 zeigt die Arbeit und Verzahnung zweier Stadtteillabore. Erstmals gegründet und entwickelt in der Großwohnsiedlung Hustadt in Bochum, stehen im Zentrum des Ansatzes eine auf Dauer angelegte Zusammenarbeit mit Nachbarschaften und der Aufbau von Beziehungen [44–46]. Im Sinne von Community-based Participatory Research, auf der auch die PGF fußt, werden Stadtteilbewohner:innen im Projekt zu Ko-Forschenden ausgebildet und wirken in allen Phasen der Forschung mit [47]. Durch eine Zusammenarbeit von Stadtteilforscher:innen über mehrere Projekte hinweg können methodische Kompetenzen und gemeinsame Arbeitsgrundlagen entwickelt werden. Und als interventionsgekoppelte Forschung vermeidet der Ansatz eine bloße Extraktion von Wissen und orientiert sich vielmehr am Selbstverständnis eingreifender forschender Praxis. Im Rahmen des Forschungsansatzes werden Forschungsprojekte zu Gesundheitszustand, Gesundheitsverständnis sowie Bedarfen an Gesundheitsförderungen und -versorgung, aber auch Forschungsprojekte aus dem Bereich der Implementierungsforschung durchgeführt. Im Stadtteillabor in Bochum wurden Interventionen der Gesundheitsförderung von Relevanz für die Community im Sinne einer partizipativen Implementierungsforschung eingeführt. Es entstanden etwa in einem Präventionsprojekt, das von der gesetzlichen Krankenversicherung (GKV) gefördert wurde, auf der Basis von partizipativer Forschung zu den Themen Stress, Diskriminierung, Sucht, Bewegung, Ernährung und Prävention 12 kultursensible Gesundheitsangebote, die u. a. dazu führten, dass Bewohner:innen erstmals an Präventionsangeboten teilnahmen. Außerdem wurde eine aufsuchende Impfkampagne initiiert, die aufgrund ihrer Kultursensibilität auch Impflinge aus anderen Stadtteilen erreichte. Faktoren, die wesentlich zum Erfolg der Intervention beigetragen haben, waren Wohnortnähe, Vertrauen und Mehrsprachigkeit [48–52]. Das Stadtteillabor in Bochum wird in der folgenden Beispielbox beschrieben und dabei wird näher auf den partizipativen Ansatz eingegangen.

In einem zweiten Stadtteillabor in Hamburg Veddel, dem Community-Health-Labor Veddel (CHL Veddel), werden die Potenziale eines Stadtteillabors mit den Möglichkeiten eines multiprofessionellen ambulanten Versorgungszentrums verschränkt und eine auf Dauer angelegte Kontaktzone zwischen der Poliklinik Veddel, der Nachbarschaft und der Hochschule für Angewandte Wissenschaften Hamburg (HAW) aufgebaut [52].^7^ Die Implementierung von Community-Forschung in diesem lokalen Kontext wird durch qualitative Interviews und Fokusgruppen mit den beteiligten Setting-Akteur:innen aus der gesundheitlichen Versorgung, der Hochschule sowie mit erfahrenen Stadtteilforscher:innen fundiert und angepasst an die vorhandenen Arbeitskonzepte, Ressourcen und soziokulturellen Praktiken in einem partizipativen Prozess entwickelt. Auf diese Weise wird u. a. sichergestellt, dass die Fachbereiche der multiprofessionellen Versorgung im Gesundheitszentrum die Community-Forschung als Bereicherung der eigenen Arbeit aufnehmen und Ergebnisse und Vertiefungswissen zu lokalen gesundheitlichen Zusammenhängen unmittelbar nutzbar machen können. Durch Implementierungsforschung wird im Rahmen von Community-based Health Care eine Versorgung weiterentwickelt, die an den Bedarfen der Nachbarschaft orientiert ist. Sie ermöglicht es, Belastungsfaktoren fundiert zu erheben und das „situierte Wissen“ von Anwohner:innen als wertvolle Ressource für Gesundheitsförderung und Versorgung zu nutzen [53]. Auch das Stadtteillabor in Hamburg wird im folgenden Beispiel ausführlich beschrieben.

#### Beispiel 1: Das Stadtteillabor Bochum als Multiple Use und die Notwendigkeit einer Verschränkung mit permanenten Versorgungstrukturen

Das Stadteillabor Bochum wurde 2016 als langzeitbezogene, partizipative Gesundheitsforschunginfrastruktur gegründet. 2019 erfolgten die ersten partizipativen Schulungen und Forschungen im Rahmen eines GKV-geförderten Präventionsprojektes, an die sich seitdem kontinuierlich weitere Forschungen in den Themenfeldern Wohnen und Digitalisierung als auch Lehrforschungsprojekte mit Studierenden und Bewohner:innen anschlossen. Als Bochumer Ortsteil, der eine am stärksten von struktureller Benachteiligung betroffene Bevölkerung aufweist, gelten Teile der Bewohnerschaft als „Schwer-Erreichbare“ [48, 49]. Die Nachbarschaft ist zudem durch eine hohe religiöse, ethnische und nationale Diversität der Bewohner:innen geprägt, von denen etwa 90 % nicht in Deutschland geboren wurden oder Eltern haben, die zugewandert sind. Außerdem besteht eine hohe Erwerbslosigkeit und viele Bewohner:innen sind auf staatliche Transferleistungen angewiesen [49]. Ziel des Stadtteillabors ist es, gemeinsam mit Bewohner:innen eine empirische Basis kleinräumiger Daten zur Entwicklung gesundheitsfördernder Angebote für die Community zu schaffen. So begannen Bewohner:innen im Zuge forschungsgekoppelter Interventionen an Präventionsangeboten teilzunehmen, eine Tendenz, die seitdem stetig zunimmt. Damit hat das Stadtteillabor wesentlich dazu beigetragen, über partizipative Forschungsprozesse Gesundheitsinterventionen lebensweltorientiert und kontextadaptiert zu gestalten [51]. Das Thema Gesundheit wurde in Kooperation mit sozialen Trägern vor Ort im Stadtteil verankert und ein multiples Netzwerk von Setting-Akteur:innen aufgebaut, die sich an der Etablierung gesundheitsfördernder Angebote beteiligen.

Die Effekte der methodischen Herangehensweise im Sinne ihrer Aktivierung vermeintlich „Schwer-Erreichbarer“ findet Anklang über die Stadtteilgrenzen hinaus, was sich an Transferanfragen aus benachbarten Kommunen, Krankenhäusern und überall dort zeigt, wo „Schwer-Erreichbarkeit“ thematisiert wird. Im Rahmen von Transferangeboten konnten die Bochumer Stadtteilforscher:innen ihre Erfahrungen mit dem partizipativen, interventionsgekoppelten Ansatz auf ähnlich strukturierte Kontexte in Witten, Gießen und Münster übertragen. 2021 lernten die Bochumer Stadtteilforscher:innen das Konzept der Poliklinik Veddel kennen und intensivierten seitdem kontinuierlich ihre Beziehungen zu den Stadtteilforscher:innen der Poliklinik und dem Community-Health-Labor Veddel [52]. Die durch gegenseitige Besuche entstehende gemeinsame Perspektive auf krank machende Verhältnisse in beiden Stadtteilen und die damit einhergehende Thematisierung und Bewusstwerdung gesundheitlicher Ungleichheit entfalten dabei eine selbstermächtigende, solidarische Wirkung.

Die Arbeit im Stadtteillabor trug zur Sichtbarwerdung von Community-Wissen bei und zur Erhöhung der Selbstwirksamkeit, was sich u. a. darin zeigt, dass die Stadtteilforscher:innen krank machende Lebensverhältnisse zunehmend infrage stellen. Befragungen und öffentliche Ergebnisvorstellungen in Policy-Cafés brachten sie in Kontakt mit Politiker:innen und lehrten sie, Rechte einzufordern. So führten Bewohner:innen Kampagnen gegen gesundheitsbelastende Wohnverhältnisse und thematisierten diese filmisch in sozialen Medien. Die Herausforderungen des Labors liegen in fehlender Strukturförderung und in der Drittmittelabhängigkeit. Die erhoffte Verstetigung der Projekte vonseiten der Kommune bleibt aus. Aus der Erkenntnis dieses Defizits und der Notwendigkeit einer strukturell verankerten multiprofessionellen Gesundheitsversorgung heraus gründeten Stadtteilforscher:innen gemeinsam mit Studierenden, praktizierenden Psychotherapeut:innen und Gesundheitsaktivist:innen im Sommer 2025 den Verein Stadtteilgesundheitszentrum Querenburg e. V. Ziel des Vereins ist die Etablierung eines multiprofessionellen Versorgungszentrums nach dem Hamburger Vorbild, und zwar in unmittelbarer Verzahnung mit Community-Forschung. Einen ersten Schritt stellt hier die Durchführung des deutschlandweit zweiten Community Health Survey in Kooperation mit Hochschulen und Kommunen als Bedarfsanalyse und empirische Grundlage dar.

#### Beispiel 2: Das Community-Health-Labor Veddel: Community-based Healthcare strukturell entwickeln

In Hamburg-Veddel, einer von Migration geprägten Elbinsel im Zentrum Hamburgs, mit 4300 Bewohner:innen, hat die Gruppe für Stadtteilgesundheit und Verhältnisprävention e. V. 2017 das Modellprojekt Stadtteilgesundheitszentrum Poliklinik Veddel^8^ gegründet, in dem multiprofessionelle Versorgung durch die Zusammenarbeit unterschiedlicher Fachbereiche (hausärztliche Praxis, Community Health Nurses, Sozial- und Gesundheitsberatung, psychologische Beratung, Hebammen und Gemeinwesenarbeit) realisiert wird. Ein besonderer Schwerpunkt liegt auf Gesundheitsförderung und Verhältnisprävention [54]. Im Jahr 2022 hat die Poliklinik Veddel gemeinsam mit einer Gruppe von Stadtteilforscher:innen den Community Health Survey Veddel als bundesweit erste partizipative, quantitative und mehrsprachige Vollerhebung eines Stadtteils durchgeführt und in Kooperation mit der HAW Hamburg ausgewertet.^9^ Ein wichtiges Ergebnis des Survey war, dass 96 % der Befragten angaben, gerne oder überwiegend gerne auf der Veddel zu leben. In Bezug auf die zentrale soziale Determinante Wohnen gab aber über 1/3 der Befragten an, Probleme mit Schimmel zu haben [55]. Zudem zeigt sich, dass trotz im Hamburger Durchschnitt relativ geringer Mieten die Mietbelastungsquote hoch ist und 43 % der Befragten Schwierigkeiten haben, mit dem Haushaltseinkommen über die Runden zu kommen.

Im Rahmen des Citizen-Science-Preises 2023 wurde anschließend mit qualitativen Methoden zu Umgangsstrategien der Veddeler:innen mit belastenden Wohnsituationen geforscht.^10^ Ein zentrales Ergebnis der partizipativen Forschung sind teils deutliche Stresssymptomatiken und wiederkehrendes Ohnmachtserleben im Umgang mit Vermieter:innen. Als Intervention entschieden die Stadtteilforscher:innen einen Mieter:innen-Rat mit Unterstützung durch den Fachbereich Gemeinwesenarbeit der Poliklinik zu gründen.

Diese beiden Forschungen sowie die Erfahrungen mit dem Aufbau des Stadtteillabors Bochum-Hustadt bilden den Ausgangspunkt, um interventionsgekoppelte Community-Forschung strukturell in einer Verschränkung von Community-Forschung, Hochschule und Stadtteilgesundheitszentrum zu verankern. So begegnet das CHL Veddel auch einem häufig auftretenden Problem partizipativer Forschungsprojekte, welche i. d. R. als temporäre Einzelprojekte zu spezifischen Themen durchgeführt werden, wodurch die Entwicklung längerer Wirkungsketten durch iterative Prozesse von Forschung und Intervention behindert werden und der Vertrauensaufbau sowie die Bildung von Expertise bei den mitforschenden Stadtteilforscher:innen erschwert werden.

Mit dem CHL Veddel können für den Stadtteil relevante Forschungen z. B. zu gesundheitlichen Bedarfen, Gesundheitshandeln, gesundheitsfördernden Ressourcen und Fragen kontextadaptierter Implementierung von Gesundheitsinterventionen durchgeführt werden und Forschungsergebnisse direkt in die lokale Versorgung der Poliklinik Veddel einfließen. Ebenso können sich aus der Versorgungspraxis Fragestellungen ergeben, die gemeinsam mit Stadtteilbewohner:innen bearbeitet werden. Derzeit werden passende Schnittstellen, gelingende Formate und an den Arbeitsalltag sowie die Eigenlogiken einer Versorgungseinrichtung angepasste Verfahrensweisen entwickelt. Auf diese Weise wird eine bundesweit neuartige, strukturell verankerte Verbindung von gesundheitlicher Primärversorgung, Gemeinwesenarbeit und partizipativer Community-Forschung erprobt, welche *Community-based Healthcare* systematisch (weiter-)entwickelt. Dies befördert zugleich Empowerment von Nachbar:innen/Patient:innen, Austausch auf Augenhöhe, vertieftes Verständnis des Sozialraums, Ansatzpunkte für Interventionen und Gesundheitsförderung und ein Netzwerk von Multiplikator:innen [56]. Durch gemeinsam mit Community-Forscher:innen durchgeführte Lehrforschungsprojekte in Studiengängen angewandter Interventionswissenschaften entstehen gleichzeitig neue Erfahrungsräume und ein Modell für die nachhaltige Zusammenarbeit von Hochschule und Stadtgesellschaft.

## Diskussion

Partizipative Forschungsansätze in der Implementierungswissenschaft können neue Möglichkeiten eröffnen, um Innovationen in die Praxis zu bringen. Die Nutzung von partizipativen Ansätzen kann jedoch in Hinblick auf Qualität und Kontinuität der Partizipation sowie Umfang der Ko-Kreation herausfordernd sein. Die Einbindung von Personen in der Implementierungsforschung konzentriert sich, wenn partizipative Ansätze verwendet werden, häufig auf die Partizipation von Umsetzenden und seltener auf die Partizipation von Nutzenden oder Bürger:innen [27, 29, 35]. Zudem werden Machtverhältnisse in der Implementierungsforschung zumeist weniger demokratisch bestimmt und die Mitbestimmung der Akteur:innen, die in dem beforschten Bereich aktiv sind oder betroffen sind, ist häufiger und auf bestimmte Phasen des Forschungsprozesses beschränkt [27]. Weiterhin ist die Durchführung von partizipativer Forschung in der Implementierungsforschung zum Teil erschwert durch die Vielzahl der unterschiedlichen Prioritäten der verschiedenen Stakeholder, die im Forschungsprozess geeint werden sollten [23].

Eine Limitation unserer Übersicht ist ihre rein exemplarische Darstellung einzelner Forschungsprojekte, die partizipative Ansätze verfolgt haben und einen Bezug zur Implementierungswissenschaft haben. Die hier vorgestellten Projekte verorten sich überwiegend in der PGF, ohne systematisch auf die Implementierungsforschung Bezug zu nehmen, obwohl Fragestellungen untersucht werden, die aus dem Feld der Implementierungsforschung kommen.

In der Implementierungsforschung im deutschsprachigen Raum beginnt jedoch aktuell die Diskussion um partizipative Ansätze, deren Mehrwert, Qualität und Umfang [1, 38]. Zunehmend fordern und fördern außerdem Fördermittelgeber die Beteiligung von Patient:innen an Forschungsprozessen [1, 5]. Wir konnten (noch) kein partizipatives Forschungsprojekt finden, das systematisch kontextbezogene Faktoren oder Outcomes unter der Verwendung von TMFs erfasst hat, wie sie in der Implementierungswissenschat weitverbreitet sind. Eine partizipative Implementierungsforschung, wie sie im angloamerikanischen Raum bereits in Ansätzen Verbreitung gefunden hat, steckt in Deutschland noch in den Kinderschuhen. Dennoch bieten partizipative Projekte aus dem Bereich der Implementierungsforschung einen Einblick in die Möglichkeiten, die sich durch die PGF für die Implementierungsforschung ergeben. [7, 13].

## Fazit

Partizipative Gesundheitsforschung kann die Implementierungsforschung in Hinblick auf gesundheitliche Chancengerechtigkeit stärken, da sie Menschen in besonders vulnerablen Situationen in den Forschungsprozess einbezieht. Dies führt sowohl zu besserer Evidenz hinsichtlich ihrer Bedarfe und Barrieren als auch zur Verbesserung der Akzeptanz, Erreichbarkeit, Reichweite und Lebensweltorientierung der Intervention [32]. So ermöglicht die Verbindung von PGF und Implementierungswissenschaft die Schaffung von Synergien und somit die nachhaltige Verbesserung der Gesundheitsversorgung [28].

Die Einbindung von partizipativen Ansätzen in die Implementierungsforschung kann neue Möglichkeiten eröffnen, relevante und passgenaue Forschungsziele und Ergebnisindikatoren mit Betroffenen und Umsetzenden zu entwickeln, Forschungsmethodik praktikabel zu gestalten und den Prozess der Implementierungsforschung bereits mit Netzwerkbildung, Empowerment und Kapazitätenaufbau zu verbinden, und so zur Akzeptanz und Nachhaltigkeit der Umsetzung beitragen.

## Supplementary Information

Englische Übersetzung des Artikels

## Footnotes

1 https://www.icphr.org/ (Zugriff am 16.03.2025).

2 http://partnet-gesundheit.de/ (Zugriff am 16.03.2025).

3 https://dnvf.de/gruppen/ag-partizipative-versorgungsforschung.html (Zugriff am 16.03.2025).

4 https://www.rheuma-liga.de/unser-einsatz/rheumaforschung/partizipative-forschung (Zugriff am 28.01.2025).

5 https://www.capital4health.fau.de/ (Zugriff am 28.01.2025).

6 https://www.partkommplus.de/teilprojekte/index.html (Zugriff am 28.01.2025).

7 https://www.poliklinik1.org/ (Zugriff am 28.01.2025).

8 https://www.poliklinik1.org/ (Zugriff am 28.01.2025).

9 https://www.veddel-wie-gehts.de/ (Zugriff am 28.01.2025).

10 https://www.citizenscience-wettbewerb.de/autorin/community-health-gesundheit-und-wohnen-auf-der-veddel (Zugriff am 28.01.2025).
