# Supplementary material for: Participation and co-creation in implementation research
Source: Bundesgesundheitsblatt Gesundheitsforschung Gesundheitsschutz. 2025 Jun 19;68(7):728–37. [Article in German] doi: 10.1007/s00103-025-04085-7 (PMC12254173; doi:10.1007/s00103-025-04085-7)
Supplement: Supplementary file 1 — Englische Übersetzung des Artikels [file 103_2025_4085_MOESM1_ESM.docx]

*This is an English translation of the article: "Partizipation und Ko-Kreation in der Implementierungsforschung" (Bundesgesundheitsblatt 7/2025). The responsibility for the translation lies solely with the authors. Please note that only the original German-language article should be cited.*

Article type: Narrative overview

**Participation and co-creation in implementation research**

Anja Zscheppang^1^, Christiane Falge^2^, Silke Betscher^3^, Anna Köster-Eiserfunke^3,4^, Jonas Fiedler^4^, Claudia Czernik^1^, Claudia Hoevener^5^, Anna Kuehne^1^

^1^ University Chair in Public Health, Centre for Evidence-based Health Care (ZEGV), University Hospital and Faculty of Medicine Carl Gustav Carus at TU Dresden, Dresden, Germany

^2^ Professorship for Health and Diversity, Urban District Laboratory Bochum, Bochum University of Applied Sciences, Bochum, Germany

^3^ Professorship for Community Work, Community Development and Macro Social Work Department of Social Work, HAW, Hamburg, Germany

^4^ Poliklinik Veddel, Hamburg, Germany

^5^ Alice Salomon Hochschule, University of Applied Sciences, Berlin, Germany

**Correspondence address**:

Prof. Dr Anna Kuehne M.Sc. M.Sc.

University Chair in Public Health

Centre for Evidence-based Healthcare (ZEGV)

University Hospital and Faculty of Medicine Carl Gustav Carus at TU Dresden Fetscherstr. 74

01307 Dresden

Germany

Email: oeffentliche.gesundheit@tu-dresden.de

# Abstract

The aim of implementation research is to transfer evidence-based interventions from research into practice. Successful transfer requires acceptance of the interventions by users and implementers. Research processes are increasingly focussing on participatory approaches in which various stakeholders are involved in research and implementation, thereby increasing the acceptability of interventions. Depending on the intervention, participation may draw from users, research, care providers, or policy and funding bodies. Citizens and patients play a crucial role in participatory processes. Participatory implementation research is a collaborative approach that combines implementation research with the co-creation of knowledge by systematically involving participants in the research process and beyond. In this way, user-centred, tailor-made, lifeworld-oriented interventions in health promotion and healthcare can be scientifically developed under real-life conditions and permanently transferred into real-world practice. This review article examines the status of participatory implementation research in Germany and outlines the concepts and framework of participatory implementation research. To this end, projects from the fields of patient care, health promotion and the community setting are described. Two examples of the long-term participation of citizens of a neighbourhood in research conducted in the urban district laboratories in Bochum and Hamburg show how continuous participation and co-creation in implementation research for prevention, health promotion and health care can succeed.

**Keywords***:*

Implementation science; community-based participatory research; evidence-based practice; capacity building; public health

# Introduction

**Participatory approaches in implementation research enable the joint development of relevant research objectives and indicators, increase the practicability of the methodology and promote networking, empowerment and capacity building. The integration of participatory approaches can strengthen the implementation process and contribute to sustained adoption. We provide an overview of the approaches of implementation research, participatory research, as well as the potential of combining both research approaches. We also present examples of participatory implementation research in Germany.**

# What is implementation research?

While the effectiveness of numerous public health measures has been scientifically proven, the transfer of these measures from research into practice often takes many years, or adoption fails under real-life conditions [1]. This lack of integration of evidence-based interventions into practice leads to underuse, overuse and misuse [2]. The translation of scientific findings into practical implementation is the research field of implementation science [3,4].

Implementation research investigates how interventions whose basic effectiveness has already been proven can be appropriately implemented in practice [3,5,6]. The focus is on identifying factors that promote or inhibit implementation. A key focus is also on sustainability, i.e. the long-term adoption of interventions beyond the implementation phase [3,5,6]. The implementation-related factors that are analysed in implementation research include [7]:

- Acceptability and appropriateness of the intervention,
- Feasibility, adoption and penetration,
- Fidelity of the original intervention,
- Sustainability, and
- Cost of the intervention.

Various theories, models and frameworks (TMFs) are used in implementation research to systematically analyse implementation processes, framework conditions and outcomes [8]. The "Consolidated Framework for Implementation Research" (CFIR) represents an approach to systematically record context-related factors [9]. The CFIR framework model summarises potential factors for transfer in five dimensions [9]: intervention characteristics; inner setting (within the organisation); outer setting (outside the organisation, e.g. legal framework and market situation); characteristics of individuals, and processes during implementation.

The article by Weishaar et al. in this special issue provides a comprehensive overview of implementation science in the public health sector in Germany as well as insights into other TMFs and application examples.

Other characteristics of implementation research include interdisciplinary collaboration, identification of context-specific success factors, exploration of capacity-building opportunities for continuation, network development, iterative improvement and continuous learning, and testing of options to scale [5]. Involving researchers, caregivers, users, and policy and funding stakeholders in implementation research can be crucial to increase the relevance and acceptability of the intervention [5,7].

# What is participatory research and co-creation

Stummer (2023) describes how implementation research can benefit from participatory approaches: "By fostering partnerships between researchers, practitioners, policy makers and communities, implementation research can facilitate the exchange of knowledge, experience and resources, ultimately leading to more effective interventions and improvements in public health." [5].

The integration of participatory health research (PHR) approaches can complement implementation science in the areas of health promotion and health care. It also provides a framework for the structured involvement of different stakeholders in the research process. PGF helps to bridge the gap between research findings and practical application, ensuring that interventions are not only evidence-based, but also contextualised and sustainable [10]. User involvement makes it easier to improve the health of groups that are often particularly poorly reached by health promotion, health care and research [5,11,12].

PGF is characterised by the direct involvement in the research process of people whose working or living conditions, health status, or needs are the subject of the research [13,14]. Participation can involve all people affected by the research or the object of the research. Participation includes funders, policy makers, practitioners, e.g. carers, doctors and other health professionals, patient representatives, or citizens [15,16]. For implementation science, both the target group of the intervention and the implementers are relevant.

In PGF, participation is understood as a research collaboration that ideally spans the entire research process. It begins with the joint definition of the research question, followed by the joint decision on methods, data collection and data analysis. PGF also includes the joint interpretation of results and development of recommendations [14]. Participatory research is thus not a commitment to a specific form of data collection - i.e. no commitment to qualitative or quantitative methodology or specific TMFs - but rather an approach to the research process [14,17,18]. This approach is characterised by research that is embedded in the local context, with participatory decision-making and a focus on the collective research process in which at least part of the decision-making power is transferred from the researchers to the other stakeholders involved [14,19].

There are various schools and orientations of PGF, which use different terms (e.g. action research, community-based participatory research), but all of which are generally characterised by cooperation between stakeholders affected by the topic under research (users, citizens, staff, practitioners, etc.) and researchers [13]. Another common feature is the connection between the generation of knowledge and the development of new options for action or interventions that improve the working or living conditions of those involved [14]. PGF involves co-creation, i.e. the joint creation of knowledge as part of a joint dialogue-based research process. This is often managed by a research team consisting of representatives from all participating groups [20,21,22]. Co-creation serves the collaborative and equal generation of knowledge. The participants are willing to share their knowledge, skills and resources. The intervention is jointly planned, designed, tested, and implemented [23]. Co-creation aims to reduce inequalities and can facilitate sustainable change [19].

Direct participation in the research process enables the development of appropriate, context-specific and locally accepted research and interventions. In addition, the PGF is a collaborative endeavour that aims to build networks, enable mutual learning, develop relevant recommendations and thus contribute to sustainability [14,22].

Participatory research processes can differ in regards to the quality of participation, the duration of participation and the level of participation. With regard to the quality of participation, various quality criteria have been established, including the inclusion of the system in which the research takes place and the appreciation of the process as a collective learning process [19]. Stakeholder participation can vary throughout the research process and for different groups. In Wright's staged model of participation, precursors to participation, such as information, consultation or involvement of stakeholders affected by the topic under research, are distinguished from actual participation in the form of co-determination, partial decision-making authority, decision-making power or even self-management [21]. The International Collaboration for Participatory Health (ICPHR) and the Network for Participatory Health Research (PartNet) offer a wide range of materials on quality characteristics, process design and networking.^[[1]](#footnote-1)^^[[2]](#footnote-2)^

Participatory research improves capacity building, i.e. structures and competences, for implementation as well as the sustainability and quality of interventions [24,25].

# How can participatory research complement implementation research?

"If we want more evidence-based practice, we need more practice-based evidence." [26]

The aim of producing scientific findings under real-life conditions unites PGF and implementation research [27]: Participatory health research emphasises the involvement of those affected and those implementing the project, with the aim of taking into account different perspectives within the research. This increases the suitability for the context, and the acceptability and sustainability of the intervention. Implementation science pursues the same goal. It also aims to put findings into practice in a context-adapted and long-term manner [23,27,28].

Some authors describe participatory research approaches as a central methodological basis for implementation research, which helps to increase local effectiveness and generate more easily generalisable evidence [29-31]. In Germany, there is a working group on participatory health services research within the German Network for Health Services Research (DNVF), which examines participatory methods of implementation science ^[[3]](#footnote-3)^.

## Practical realisation of participatory health research in implementation science

Participatory implementation research is described internationally as an approach within implementation research. It is a collaborative research approach that involves researchers, stakeholders and implementers to integrate evidence-based interventions into practice in a context-adapted manner. The participatory approach aims to improve the health of individuals in their communities and reduce health inequalities [11,32]. In contrast to traditional top-down models, participatory approaches in implementation research emphasise the co-production of knowledge through a relational and non-linear process. PGF can improve the fit of implementation strategies by enabling immediate feedback and iterative improvements that are critical for successful implementation in different settings [11]. Participatory approaches can be applied throughout the implementation research process [11]. Participatory implementation research considers the design of networks and the development of information formats tailored to stakeholder needs [11]. By incorporating PGF methods, implementation research can shorten the time between scientific confirmation of the effectiveness of interventions and their implementation in practice [28].

## Scope and variations of participatory health research in implementation research

In fact, numerous authors describe participatory approaches in implementation research [10,28,23,32-34]. These approaches differ in terms of a) the stakeholder involved, b) the depth of co-determination (i.e. the level of participation and the associated transfer of power) as well as c) in the length and duration of involvement [27,23,29,35,36].

The involvement of those being researched is often project-specific and is sometimes limited to individual aspects of the research process. Participatory research in implementation science can be used both in research planning - for example in determining the (health) outcome - and in determining the research design, intervention design and data interpretation. It is possible in all phases of the research process or even beyond in the form of long-term research partnerships [10,11,27,23,37]. Some research partnerships are topic-specific and focus on a particular research question, while long-term, open-topic partnerships between universities, practitioners and citizens allow for joint prioritisation and development of implementation strategies [37]. A particularly extensive and long-term form of PGF is "Community-Engaged Dissemination and Implementation Research" (CEDI) [31], which is characterised by long-term partnerships between researchers and communities, ongoing community engagement and the joint development of projects [31,36]. In the US Clinical and Translational Science Institutes, community engagement in the sense of a long-term, ongoing partnership on an equal footing is already taking place and is increasingly being used for implementation research [34].

To summarise, the application of PGF in implementation research can involve different stakeholders, be used in different phases of the research process, and involve different levels of participation [38] (Fig. 1).

# Examples of participatory approaches in implementation research in Germany

There are many projects in Germany that use participatory approaches in implementation research. The participatory research approach or co-creation and/or the identification with the field of implementation research is not always explicitly stated in the projects. In the following, we present selected projects that:

1. include participatory research methods in the sense of co-creation, with the transfer of decision-making power to stakeholder affected by the topic under research, and
2. which dealt with a subject matter typical of implementation research such as research into the integration of an intervention whose effectiveness has already been proven in a specific context/for a specific group.

In the following, individual participatory projects from the field of implementation science are discussed as examples illustrating the range of participatory research in Germany. The research projects differ in terms of the quality of participation, its ongoing nature, and the intensity of participation. They address different groups and use different research methods. What all projects have in common is that they investigate the transfer into practice of an intervention with proven health benefits. In the literature, we found no example of a project in Germany in which participatory research methods were applied and structured TMFs of implementation research were used.

## Patient participation in implementation research in medical care

Participatory research approaches can be found in various specialised areas of medical care. One important example to highlight is the approach by the German Rheumatism League, which the organisation promotes systematically.^4^ Their approach could be particularly suitable for implementation research projects as the lived experience of patients is examined in relation to their care and the results are important for patients in practice.^[[4]](#footnote-4)^ There are also approaches for systematically involving patients and citizens and practitioners. Participation takes place in form of patient advisory boards or citizen forums and focuses on study designs, transfer into practice, and dissemination of results [39].

## Participation in health promotion using the example of physical activity

*Capital4Health* – “Capabilites for active lifestyle: An interactive knowledge-to-action research network for health promotion" is a research network that focusses on researching and developing opportunities for interventions promoting a healthy lifestyle throughout the entire lifespan within sub-projects. These sub-projects pursue participatory approaches by involving stakeholders delivering the intervention as well as the target population groups in the research process^[[5]](#footnote-5)^. By means of "cooperative planning", different age groups in different settings are actively involved in the development of interventions and concrete action plans to promote physical activity in so-called planning groups [40,41].

The "Health.edu" sub-project (2015-2018) aimed to sustainably develop pupils' sports-related health literacy. In cooperative planning groups at four schools in the physical education setting, physical education teachers, school management, educators, and students were involved in a series of meetings (over a period of 12 to 18 months) to implement a curriculum in physical education. In parallel, meetings were also held with lecturers, university staff, coordinators of the teacher training phases, academics and students in the physical education teacher education setting. The results show that the intervention in form of physical education lessons to strengthen pupils' health literacy was successful and achieved significantly better results than in the control schools. The participants considered the "cooperative planning" method as a "rewarding process" that took place in an appreciative setting. By involving the pupils, their views on physical education and thus their expertise could be taken into account in the planning of interventions. In this way, the project adapted physical education as a physical activity-promoting health intervention in terms of participatory implementation research and also achieved a greater understanding of health and physical activity in the research process [41]. Aspects of acceptability, feasibility and adoption were researched as part of the project.

## Participation in the community - municipal health strategies

Under the umbrella of the PartKommPlus “Forschungsverbund für gesunde Kommunen” [Research Network for Healthy Communities"], seven participatory research projects were carried out with the aim of supporting healthy lifestyles through municipal or company programmes.^[[6]](#footnote-6)^ In some of the funded projects, health and public health interventions whose effectiveness had already been proven, were combined with implementation research approaches to identify barriers and enablers for implementation. In addition, user-centred interventions were developed that were adapted to the real world.

As one of the PartKommPlus projects, the participatory research project GESUND! investigated health promotion for and with people with learning difficulties [42]. It consisted of several components, each of which adapted health promotion measures to the target group of people with learning difficulties. Interventions in the areas of healthy eating, reducing noise pollution, health promotion in workplaces for people with learning difficulties and health education were researched. In some cases, these were established in the long term as part of participatory research projects that used both quantitative surveys and qualitative methods and PhotoVoice methodology [41].

Another project from the PartKommPlus network is the KEG project (Kommunale Entwicklung von Gesundheitsstrategien: Science and Practice in Dialogue), a participatory project that explores how the development and implementation of municipal health strategies can succeed [43]. Health promotion practitioners and young residents of the neighbourhood came together and used the "Appreciative Inquiry" method to conduct interviews with stakeholders and residents of the neighbourhood in a qualitative research process. Within this framework, the research-practice partnership developed and evaluated a framework to conceptualise and implement local health strategies adapted to the local context [43].

## Ongoing participatory research in the context of neighbourhood laboratories

The methodological and conceptual approach of a district laboratory is characterised by a close relationship between a university, practitioners and neighbourhoods. Figure 2 shows the work and interlinking of two urban district laboratories. First founded and developed in the large housing estate of Hustadt in Bochum, Germany, the focus of the approach is on long-term cooperation with neighbourhoods and on building relationships [44-46]. In the spirit of community-based participatory research, on which the PGF is also based, neighbourhood residents are trained as co-researchers and participate in all phases of the research project [47]. Through cooperation between neighbourhood researchers across several projects, methodological skills and common working principles can be developed. Importantly, as intervention-linked research, the approach avoids the mere extraction of knowledge and is instead orientated towards the self-image of interventional research practice. As part of the research approach, research projects are carried out on the state of health, understanding of health, and the need for health promotion and care, as well as research projects in the field of implementation research. In the urban district laboratory in Bochum, health promotion interventions of relevance to the community were introduced in the framework of participatory implementation research. For example, in a prevention project funded by the statutory health insurance (GKV), 12 culturally sensitive health programmes were developed on the basis of participatory research into the topics of stress, discrimination, addiction, exercise, nutrition and prevention. This, among other things, led to residents taking part in prevention programmes for the first time. In addition, an outreach vaccination campaign was initiated, which also reached non-vaccinated people from other neighbourhoods due to its cultural sensitivity. Factors that contributed significantly to the success of the intervention were proximity to the neighbourhood, trust and multilingualism [48-52]. The neighbourhood laboratory in Bochum is described below and the participatory approach is discussed in more detail.

In a second district laboratory located in Hamburg Veddel, the Community Health Laboratory Veddel (CHL Veddel), the advantages of a district laboratory are combined with the services of a multi-professional outpatient care centre and ongoing collaboration exists between the Veddel Polyclinic, the neighbourhood and the Hamburg University of Applied Sciences (HAW) [52]^[[7]](#footnote-7)^ . The implementation of community research in this local context is based on qualitative interviews and focus groups with the participating setting-specific stakeholders from healthcare services, the university and experienced neighbourhood researchers. It is developed in a participatory process adapted to the existing work concepts, resources and socio-cultural practices. This ensures, among other things, that the specialist areas of multi-professional care in the health centre can take up community research as an enrichment of their own work and make the results and in-depth knowledge of local health contexts directly usable. Through implementation research, care that is oriented towards the needs of the neighbourhood is further developed within the framework of community-based health care. It makes it possible to identify stress factors and utilise the "situated knowledge" of residents as a valuable resource for health promotion and care [53]. The neighbourhood laboratory in Hamburg is also described in detail below.

*Example 1: The Bochum urban district laboratory as multiple use and the necessity of interlinking with permanent care structures*

The “Stadteillabor Bochum” was founded in 2016 as a piece of long-term, participatory health research infrastructure. In 2019, the first participatory training and research program was carried out as part of a GKV-funded prevention project, which has since been followed by further research in the fields of housing and digitalisation. In addition, teaching and research projects with students and residents have taken place. As a district of Bochum whose population is disproportionately affected by structural disadvantage, a subset of residents is considered "hard to reach" [48,49]. The neighbourhood is also characterised by a high level of religious, ethnic and national diversity among its residents, around 90% of whom were not born in Germany or have parents who have immigrated. There is also a high level of unemployment and many residents are dependent on government benefits. [49] The aim of the neighbourhood laboratory is to work with residents to create an empirical basis of small-scale data for the development of health-promoting services for the community. As a result, residents began to participate in prevention programmes as part of research-linked interventions, a trend that has been steadily increasing ever since. The urban neighbourhood laboratory has thus made a significant contribution to designing health interventions that are considerate of lived experiences and are context-adapted through participatory research processes [51]. The topic of health was anchored in the neighbourhood in cooperation with local social institutions. A network of stakeholders from the setting was established who are involved in the establishment of health-promoting services.

The effects of the methodological approach in terms of activating supposedly "hard-to-reach" populations have been well received beyond the district boundaries, as can be seen from transfer requests from neighbouring municipalities, hospitals and wherever "hard-to-reach" groups are addressed. As part of transfer programmes, the Bochum district researchers were able to transfer their experience with the participatory, intervention-linked approach to similarly structured contexts in Witten, Gießen and Münster. In 2021, the Bochum-based neighbourhood researchers became familiar with the concept of the Veddel Polyclinic and have since intensified their relationships with the neighbourhood researchers at the Polyclinic and the Veddel Community Health Laboratory on an ongoing basis [52]. The shared perspective on disease-causing conditions in both neighbourhoods that emerges through mutual visits and the associated examination and awareness of health inequality have a self-empowering, solidarity-based effect.

The work in the neighbourhood laboratory contributed to making community knowledge visible and to increasing self-efficacy. This is reflected, among other things, in the fact that the neighbourhood researchers are increasingly questioning living conditions that cause illness. Surveys and public presentations of results in policy cafés brought them into contact with politicians and taught them to demand rights. Residents led campaigns against unhealthy living conditions and discussed these on social media. The challenges of the lab lie in the lack of structural support and dependence on third-party funding. The hoped-for provision of the projects on the part of the municipality has not materialised. Recognising this deficit and the need for structurally anchored multi-professional healthcare, district researchers, together with students, practising psychotherapists and health activists, founded the association Stadtteilgesundheitszentrum Querenburg e.V. in the summer of 2025. The aim of the association is to establish a multi-professional healthcare centre based on the Hamburg model and directly linked with community research. A first step in this direction is the implementation of Germany's second Community Health Survey in cooperation with universities and local authorities to serve as a needs assessment and an empirical basis for further work.

*Example 2: The Veddel Community Health Laboratory: structurally developing community-based health care*

In Hamburg-Veddel, an Elbe island in the centre of Hamburg that is characterised by migration and has 4,300 residents, the ”Gruppe für Stadtteilgesundheit und Verhältnisprävention e.V.” founded the model project “Stadtteilgesundheitszentrum Poliklinik Veddel”^[[8]](#footnote-8)^ in 2017. Here, multi-professional care is provided through cooperation between different specialist areas (general practice, community health nursing, social and health counselling, psychological counselling, midwifery, and community work). A particular focus is on health promotion and conditional prevention [54]. In 2022, the Veddel Polyclinic, together with a group of district researchers, conducted the Community Health Survey Veddel as the first participatory, quantitative and multilingual full survey of a district in Germany and evaluated it in cooperation with HAW Hamburg.^[[9]](#footnote-9)^ An important result of the survey was that 96% of respondents stated that they liked or mostly liked living in Veddel. However, with regard to the key social determinant of housing, over one third of respondents stated that they had problems with mould [55]. It was also apparent that despite relatively low rents on average in Hamburg, the rent burden was high and 43% of respondents had difficulties making ends meet with their household income.

As part of the research prize “Citizen Science Award 2023”, qualitative methods were then used to research the Veddel residents' strategies for dealing with stressful housing situations.^[[10]](#footnote-10)^ A key result of the participatory research is the clear symptoms of stress and recurring feelings of powerlessness when dealing with landlords. As an intervention, the neighbourhood researchers decided to set up a tenants' council with the support of the Polyclinic's community work department.

These two research projects and the experience gained with the establishment of the Bochum-Hustadt urban district laboratory form the starting point for structurally anchoring intervention-linked community research in an interlinking of community research, the university and the district health centre. In this way, the CHL Veddel also addresses a common problem of participatory research projects, which are usually carried out as temporary individual projects on specific topics. This hinders the development of longer chains of impact through iterative processes of research and intervention and makes it difficult to build trust and expertise among the participating district researchers.

With the CHL Veddel, research relevant to the neighbourhood can be conducted, e.g. on health needs, health behaviour, health-promoting resources and questions of context-adapted implementation of health interventions. In addition, research results can directly inform local care provision at the Veddel Polyclinic. Issues can also arise from healthcare practice that are worked on together with neighbourhood residents. Suitable interfaces, successful formats and procedures adapted to the day-to-day work and logic of a healthcare facility are currently being developed. In this way, a novel, structurally anchored combination of primary health care, community work and participatory community research is being trialled throughout Germany, which systematically (further) develops *community-based health care*. At the same time, this promotes empowerment of neighbours/patients, exchange on an equal footing, a deeper understanding of the social space, starting points for interventions and health promotion and a network of multipliers [56]. Teaching and research projects carried out together with community researchers in degree programmes in applied intervention sciences simultaneously create new spaces for experiences and a model for sustainable cooperation between the university and urban society.

# Discussion

Participatory approaches in implementation research can open up new opportunities to bring innovations into practice. However, the use of participatory approaches can be challenging in terms of the quality and continuity of participation and the extent of co-creation. Where participatory approaches are used, the involvement of people in implementation research frequently focuses on the participation of implementers and less often on the participation of users or citizens [27,29,35]. In addition, power relations in implementation research are often favouring academic researchers and the participation of stakeholders affected by the topic under research is often more limited and restricted to certain phases of the research process [27]. Furthermore, conducting participatory research in implementation research is sometimes made more difficult by the multitude of different priorities of the various stakeholders that need to be negotiated during the research process [23].

One limitation of our overview is that it merely presents case studies of individual research projects that have pursued participatory approaches and that are grounded in implementation research. The projects presented here are predominantly located in PGF without any systematic reference to implementation research, although the research questions align with the field of implementation research.

Nevertheless, in implementation research in German-speaking countries, the discussion about participatory approaches, their added value, quality and scope has started [1,38]. In addition, funding bodies are increasingly demanding and promoting the participation of patients in research processes [1,5]. We could not (yet) find an example of a participatory research project that systematically assessed contextual factors or outcomes using TMFs, which are already used in implementation science internationally. Participatory implementation research, which is already common in Anglophone countries, is still in its infancy in Germany. Nevertheless, examples of participatory projects drawn from the field of implementation research offer insights into the possibilities that PGF opens up for implementation research.

# Conclusion

Participatory health research can strengthen implementation research with regard to health equity, as it can involve people in particularly vulnerable situations in the research process. This leads to better evidence regarding their needs and barriers. It also improves the acceptability, accessibility, reach and local adaptation and relevance of the intervention [32]. In this way, the combination of PGF and implementation research enables the creation of synergies and thus the sustainable improvement of healthcare [28].

The integration of participatory approaches into implementation science can open up new opportunities to develop relevant and targeted research objectives and outcome indicators with citizens, users and implementers. It can also make research methodology practicable, and combining the process of implementation research with networking, empowerment, and capacity building contributes to the acceptability and sustainability of implementation.

Conflict of interest: A. Zscheppang, C. Falge, S. Betscher, A. Köster-Eiserfunke, J. Fiedler, C. Czernik, C. Hövener and A. Kuehne declare that there is no conflict of interest.

No studies on humans or animals were conducted by the authors for this article. The ethical guidelines stated there apply to the studies listed.

**Literature**

1. Gutt A-K, Hoben M, Roes M, Willmeroth T, Wesselborg B, Kuske S (2018) Systematische Übersetzung und Cross-Validierung definierter Implementierungsoutcomes der Gesundheitsversorgung [Systematic translation and cross-validation of defined healthcare implementation outcomes]. Zeitschrift für Evidenz, Fortbildung und Qualität im Gesundheitswesen 135-136:72-80. <https://doi.org/10.1016/j.zefq.2018.06.005>
2. Institute of Medicine Committee on Quality of Health Care (2001) In: Crossing the Quality Chasm: A New Health System for the 21st Century. National Academies Press (US) Copyright 2001 by the National Academy of Sciences. All rights reserved, Washington (DC)
3. Eccles MP, Mittman BS (2006) Welcome to Implementation Science. Implementation Science 1:1. 10.1186/1748-5908-1-1
4. Wensing M: Implementierungsforschung im Gesundheitswesen [Implementation research in the health care system]. In Pfaff H, Neugebauer EAM, Ernstmann N, Härter M, Hoffmann F (eds) (2024) Versorgungsforschung: Theorien – Methoden – Praxis, Wiesbaden, p 325-332
5. Stummer FO (2023) Implementierungsstrategien im Gesundheitswesen [Implementation strategies in healthcare]. Die 50 wichtigsten Implementierungs-Frameworks in der Praxis - eine Literaturrecherche. In: Springer Verlag
6. Rubenstein LV, Pugh J (2006) Strategies for promoting organisational and practice change by advancing implementation research. J Gen Intern Med 21 Suppl 2:S58-64. 10.1111/j.1525-1497.2006.00364.x
7. Petermann F (2014) Implementationsforschung: Grundbegriffe und Konzepte [Implementation research: basic terms and concepts]. Psychologische Rundschau 65:122–128. 10.1026/0033-3042/a000214
8. Nilsen P (2015) Making sense of implementation theories, models and frameworks. Implement. Scien. 10:53. https://doi.org/10.1186/s13012-015-0242-0
9. Damschroder LJ, Aron DC, Keith RE, Kirsh SR, Alexander JA, Lowery JC (2009) Fostering implementation of health services research findings into practice: a consolidated framework for advancing implementation science. Implementation Science 4:50. 10.1186/1748-5908-4-50
10. Leykum LK, Pugh JA, Lanham HJ, Harmon J, McDaniel RR (2009) Implementation research design: integrating participatory action research into randomised controlled trials. Implementation Science 4:69. 10.1186/1748-5908-4-69
11. Ramanadhan S, Alemán R, Bradley CD et al. (2024) Using Participatory Implementation Science to Advance Health Equity. Annu Rev Public Health 45:47-67. 10.1146/annurev-publhealth-060722-024251
12. Lobb R, Colditz GA (2013) Implementation science and its application to population health. Annu Rev Public Health 34:235-251. 10.1146/annurev-publhealth-031912-114444
13. Wright MT (2021) Partizipative Gesundheitsforschung: Ursprünge und heutiger Stand [Participatory health research: origins and current status]. Bundesgesundheitsblatt Gesundheitsforschung Gesundheitsschutz 64:140-145. 10.1007/s00103-020-03264-y
14. International Collaboration for Participatory Health Research (ICfPH) (2013) Position Paper 1: What is Participatory Health Research? In: International Collaboration for Participatory Health Research, Berlin. Accessed: 27.01.2025
15. Green LW, Mercer SL (2001) Can public health researchers and agencies reconcile the push from funding bodies and the pull from communities? Am J Public Health 91:1926-1929. 10.2105/ajph.91.12.1926
16. von Unger H (2012) Participatory Health Research: Who Participates in What? Forum Qualitative Sozialforschung / Forum: Qualitative Social Research 1310.17169/fqs-13.1.1781
17. Bär G, Hövener C, Wright MT, Saß A-C (2021) Demokratisch und emanzipatorisch – Partizipative Gesundheitsforschung hat hohes Potenzial [Democratic and emancipatory - participatory health research has great potential]. Bundesgesundheitsblatt - Gesundheitsforschung - Gesundheitsschutz 6410.1007/s00103-020-03276-8
18. International Collaboration for Participatory Health Research (ICfPH) (2020) Position Paper 3: Impact in Participatory Health Research. In: International Collaboration for Participatory Health Research, Berlin. https://www.icphr.org/uploads/2/0/3/9/20399575/icphr_position_paper_3_impact_-_march_2020__1_.pdf. Accessed: 27.01.2025
19. Greenhalgh T, Jackson C, Shaw S, Janamian T (2016) Achieving Research Impact Through Co-creation in Community-Based Health Services: Literature Review and Case Study. Milbank Q 94:392-429. 10.1111/1468-0009.12197
20. Wright M, Brito I, Cook T et al. (2013) International Collaboration for Participatory Health Research (ICPHR) (2013) Position Paper 1: What is Participatory Health Research? Version: May 2013, Berlin.
21. Wright MT, Block M, von Unger H (2010) Partizipation in der Zusammenarbeit zwischen Zielgruppe, Projekt und Geldgeber/in [Participation in the co-operation between target group, project and funder]. In: Wright MT (ed) Partizipative Qualitätsentwicklung in der Gesundheitsförderung und Prävention. Hans-Huber, Bern, p 75–92
22. Hartung S, Wihofszky P, Wright MT (Hrsg.) (2020) Partizipative Forschung. Ein Forschungsansatz für Gesundheit und seine Methoden. [Participatory research. A research approach to health and its methods]. Springer
23. Pérez Jolles M, Willging CE, Stadnick NA et al. (2022) Understanding implementation research collaborations from a co-creation lens: Recommendations for a path forward. Front Health Serv 210.3389/frhs.2022.942658
24. Nickel S, Trojan, A. (2024) Capacity building/Kapazitätsentwicklung. In: (BZgA) BZgA (ed) Leitbegriffe der Gesundheitsförderung und Prävention. Glossar zu Konzepten, Strategien und Methoden.
25. Jagosh J, Macaulay A, Pluye P et al. (2012) Uncovering the benefits of participatory research: Implications of a realist review for health research and practice. Millbank Quarterly 9:1-41.
26. Green LW, Glasgow RE (2006) Evaluating the relevance, generalisation, and applicability of research: issues in external validation and translation methodology. Eval Health Prof 29:126-153. 10.1177/0163278705284445
27. Di Ruggiero E, Edwards N (2018) The Interplay between Participatory Health Research and Implementation Research: Canadian Research Funding Perspectives. BioMed Research International 2018:1519402. https://doi.org/10.1155/2018/1519402
28. Casey M, O' Leary D, Coghlan D (2018) Unpacking action research and implementation science: Implications for nursing. J Adv Nurs 74:1051-1058. 10.1111/jan.13494
29. Estabrooks PA, Brownson RC, Pronk NP (2018) Dissemination and Implementation Science for Public Health Professionals: An Overview and Call to Action. Prev Chronic Dis 15:E162. https://doi.org/10.5888/pcd15.180525
30. Chambers DA, Norton WE (2016) The Adaptome: Advancing the Science of Intervention Adaptation. Am J Prev Med 51:S124-131. 10.1016/j.amepre.2016.05.011
31. Holt CL, Chambers DA (2017) Opportunities and challenges in conducting community-engaged dissemination/implementation research. Transl Behav Med 7:389-392. 10.1007/s13142-017-0520-2
32. Schlechter CR, Del Fiol G, Lam CY et al (2021) Application of community-engaged dissemination and implementation science to improve health equity. Prev Med Rep 24:101620. 10.1016/j.pmedr.2021.101620
33. Bailie R, Matthews V, Brands J, Schierhout G (2013) A systems-based partnership learning model for strengthening primary healthcare. Implement Sci 8:143. 10.1186/1748-5908-8-143
34. Gunn CM, Sprague Martinez LS, Battaglia TA et al. (2022) Integrating community engagement with implementation science to advance the measurement of translational science. J Clin Transl Sci 6:e107. 10.1017/cts.2022.433
35. Rapport F, Smith J, Hutchinson K et al. (2022) Too much theory and not enough practice? The challenge of implementation science application in healthcare practice. J Eval Clin Pract 28:991-1002. 10.1111/jep.13600
36. Baumann W, Farin E, Menzel-Begemann A, Meyer T (2016) Memorandum IV: Theoretische und normative Fundierung der Versorgungsforschung [Memorandum IV: Theoretical and normative foundations of health services research]. Das Gesundheitswesen 78:337-352. 10.1055/s-0042-105511
37. Chambers DA, Azrin ST (2013) Research and services partnerships: partnership: a fundamental component of dissemination and implementation research. Psychiatr Serv 64:509-511. 10.1176/appi.ps.201300032
38. Krieger T, Nellessen-Martens, G (2023) Partizipation von Stakeholdern in der Versorgungsforschung: politische Erwartungen, Nutzen und praktische Impulse [Stakeholder participation in health services research: political expectations, benefits and practical impulses]. Monitor Versorgungsforschung 1/23:58-62. <http://doi.org/10.24945/MVF.01.23.1866-0533.2478>
39. Engler J, Kuschick D, Tillmann J, Kretzschmann C, Wallacher S, Kersting C (2022) [Patient and Public Involvement in Family Medicine Research]. ZFA (Stuttgart) 98:178-183. 10.53180/zfa.2022.0178-0183
40. Gelius P, Brandl-Bredenbeck H, Hassel H et al. (2020) Kooperative Planung von Maßnahmen zur Bewegungsförderung: Neue Wege zur Erweiterung von Handlungsmöglichkeiten – Ergebnisse aus dem Forschungsverbund Capital4Health [Cooperative planning of measures to promote physical activity: New ways to expand opportunities for action - results from the Capital4Health research network]. Bundesgesundheitsblatt - Gesundheitsforschung - Gesundheitsschutz 64:187–198. https://doi.org10.1007/s00103-020-03263-z
41. Töpfer C, Ptack K, Tittlbach S, Brandl-Bredenbeck HP, Sygusch R (2020) Reflexionen zu Health.edu: Kernbefunde und Forschungsdesiderate [Reflections on Health.edu: Core findings and research desiderata]. In: Sygusch R, Brandl-Bredenbeck HP, Tittlbach S, Ptack K, Töpfer C (eds) Gesundheit in Sportunterricht und Sportlehrerbildung: Bestandsaufnahme, Intervention und Evaluation im Projekt ‚Health.edu‘. Springer Fachmedien Wiesbaden, Wiesbaden, p 355-383
42. Becker KP, Burtscher R (2019) Gemeinsam forschen – Gemeinsam lernen. Menschen mit Lernschwierigkeiten in der Partizipativen Gesundheitsforschung [Researching together - learning together. People with learning disabilities in participatory health research]. Stiftung Rehabilitationszentrum Berlin-Ost
43. Wihofszky P, Hofrichter P, Layh S, Jahnke M (2021) Transfer partizipativer Forschungsergebnisse in die Praxis: Das Beratungsinstrument Standortanalyse in der kommunalen Gesundheitsförderung [Transfer of participatory research results into practice: The counselling tool site analysis in community health promotion]. Bundesgesundheitsblatt - Gesundheitsforschung - Gesundheitsschutz 64:199-206. 10.1007/s00103-020-03273-x
44. Falge C (2018) Mit Diversity zum Gerechtigkeits-Turn: Partizipative Langzeitforschung und Lehre im Stadtteillabor Bochum [Participatory long-term research and teaching in the Bochum urban district laboratory]. Zeitschrift für Führung und Personalmanagement in der Gesundheitswirtschaft. Thema "Personelle Vielfalt" Jg. 3 I Nr. 210.17193/HNU.ZFPG.03.02.2017-01
45. Falge C (2018) Migration und Gesundheit. Ein exemplarisches Beispiel [Migration and health. An exemplary case]. impulse für Gesundheitsförderung 2, 09-10
46. Falge C (2021) Medizinethnologie im Anwendungskontext: theoretische und methodische Orientierungen im Stadtteillabor Bochum [Medical anthropology in the context of application: theoretical and methodological orientations in the urban district laboratory Bochum] In: (Eds.) JSHZ (ed) Migration und Gesundheit Hogrefe Verlag, p 397-405
47. Wallerstein NB, Duran B (2006) Using community-based participatory research to address health disparities. Health Promot Pract 7:312-323. 10.1177/1524839906289376
48. Stadt Bochum (2023) Sozialbericht 2021, Bochum [Social Report 2021, Bochum]. In: Basisgesundheitsbericht und Fachplan Gesundheit
49. Falge C, Betscher, S, Geldermann, A, Jünger, S (2025) Die Rede von der Schwererreichbarkeit dekonstruieren: die digitale Diversitätslücke bei Gesundheitsinformationen als Hürde für Prävention und Gesundheitsförderung [Deconstructing the talk of hard-to-reach: the digital diversity gap in health information as a barrier to prevention and health promotion]. In: F. Fischer & K. Wrona (Eds.) (ed) Technologiegestützte Ansätze in der Community-basierten Prävention und Gesundheitsförderung. Springer-Verlag GmbH
50. Stadt Bochum (Hg.) (2024) Demografiebericht 2024 [Demography Report 2024]
51. Falge C, Betscher S (2024) Gesundheitliche Verhältnisse verändern durch Community Forschung im Bochumer Stadtteillabor [Changing health conditions through community research in the Bochum neighbourhood laboratory]. Public Health Forum 32:43-45. doi:10.1515/pubhef-2023-0145
52. VdÄÄ (2015) Poliklinik - Eine Konzeptzeitschrift - Soziale Determinanten von Gesundheit [Poliklinik - A concept journal - Social determinants of health] In: Zeitschrift für eine soziale Medizin. p 14-16
53. Haraway DJ (1995a) Situiertes Wissen. Die Wissenschaftsfrage im Feminismus und das Privileg einer partialen Perspektive [Situated knowledge. The Science Question in Feminism and the Privilege of a Partial Perspective]. In: (Hrsg.) d (ed) Die Neuerfindung der Natur. Primaten, Cyborgs und Frauen. Campus, Frankfurt am Main u.a., p 73–97
54. Filmar T, Schlegel K, Waidhas L et al. (2024) Interprofessionelle Primärversorgungszentren als Lösungsansatz für die Versorgungsprobleme der Zukunft – am Beispiel der Poliklinik Veddel [Interprofessional primary care centres as a solution for the care problems of the future - using the example of the Veddel Polyclinic]. In: Gesundheitswesen aktuell 2024. Barmer, p 168–187
55. World Health Organisation (2028) WHO housing and health guidelines. In, Geneva.
56. Betscher S, Falge C, Ahmad I et al. (2025) Intervention-linked Community Research - An underestimated method of community organising? Community Organising

***Figure 1****:* Visualisation of participation opportunities in participatory health research: Participation of different stakeholders, at different levels of participation, during the research process (authors’ own work based on Krieger T, Nellessen-Martens, G (2023) [38] and Wright M (2021) [12]). The levels of participation according to Wright M (2021) are shown on the left. The different stages of the research process are shown in the coloured columns. The coloured dots each represent a stakeholder. These can be entered multiple times in the table to make it clear who is involved at which level of participation in the research process and when.

***
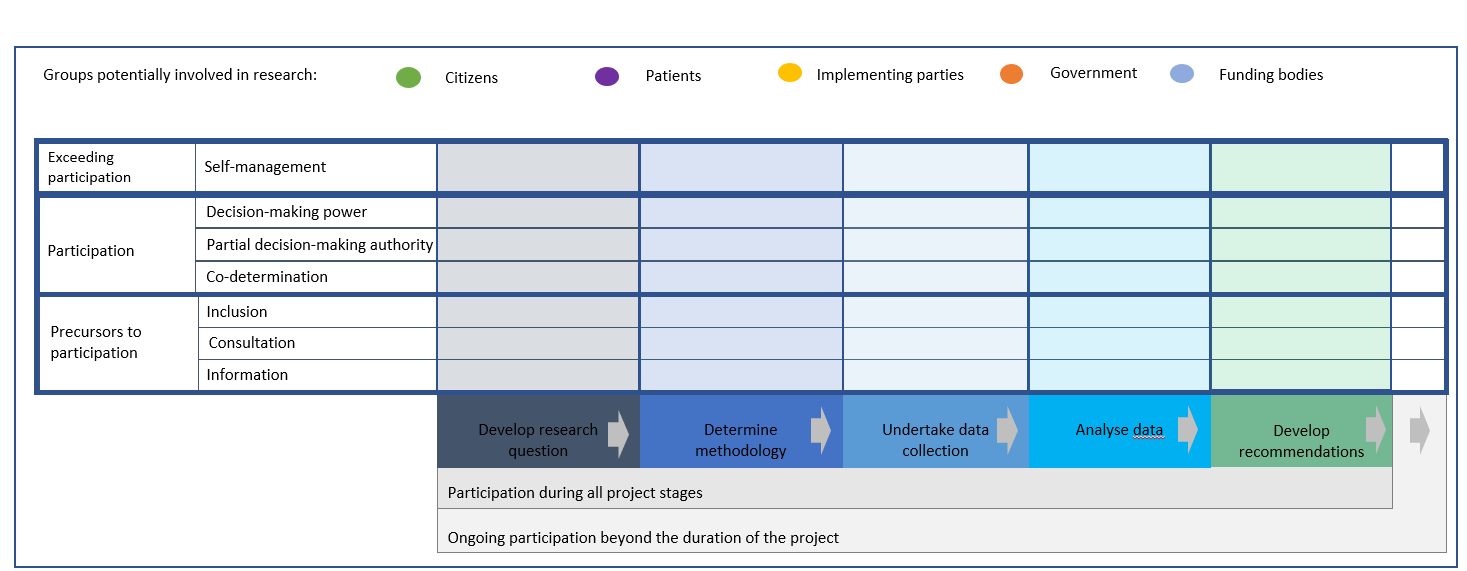
***

***Figure 2***: Structure of the Bochum and CHL Veddel district laboratories. Authors’ own work.


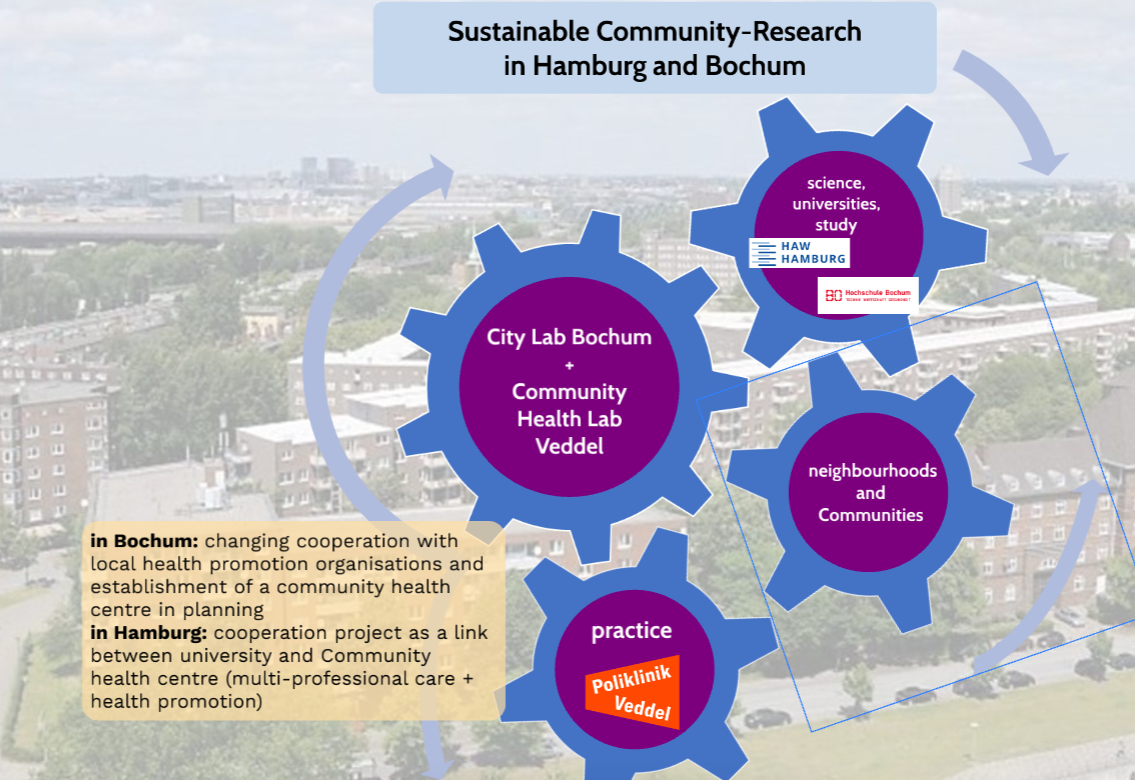


1. <https://www.icphr.org/>[accessed 16 March 2025]. [↑](#footnote-ref-1)
2. <http://partnet-gesundheit.de/>[accessed 16 March 2025]. [↑](#footnote-ref-2)
3. <https://dnvf.de/gruppen/ag-partizipative-versorgungsforschung.html>[accessed 16 March 2025]. [↑](#footnote-ref-3)
4. https://www.rheuma-liga.de/unser-einsatz/rheumaforschung/partizipative-forschung [accessed 28 January 2025]. [↑](#footnote-ref-4)
5. https://www.capital4health.fau.de/ [accessed 28 January 2025]. [↑](#footnote-ref-5)
6. https://www.partkommplus.de/teilprojekte/index.html [accessed 28 January 2025]. [↑](#footnote-ref-6)
7. https://www.poliklinik1.org/ [accessed 28 January 2025]. [↑](#footnote-ref-7)
8. https://www.poliklinik1.org/ [accessed 28 January 2025] [↑](#footnote-ref-8)
9. https://www.veddel-wie-gehts.de/ [accessed 28 January 2025] [↑](#footnote-ref-9)
10. https://www.citizenscience-wettbewerb.de/autorin/community-health-gesundheit-und-wohnen-auf-der-veddel [accessed 28 January 2025] [↑](#footnote-ref-10)
